# Activation of a Chimeric Rpb5/RpoH Subunit Using Library Selection

**DOI:** 10.1371/journal.pone.0087485

**Published:** 2014-01-29

**Authors:** Bettina Sommer, Ingrid Waege, David Pöllmann, Tobias Seitz, Michael Thomm, Reinhard Sterner, Winfried Hausner

**Affiliations:** 1 Institute of Biophysics and Physical Biochemistry, University of Regensburg, Regensburg, Germany; 2 Institute of Microbiology and Archaea Center, University of Regensburg, Regensburg, Germany; University of Florida, United States of America

## Abstract

Rpb5 is a general subunit of all eukaryotic RNA polymerases which consists of a N-terminal and a C-terminal domain. The corresponding archaeal subunit RpoH contains only the conserved C-terminal domain without any N-terminal extensions. A chimeric construct, termed *rp5H*, which encodes the N-terminal yeast domain and the C-terminal domain from *Pyrococcus furiosus* is unable to complement the lethal phenotype of a yeast *rpb5* deletion strain (Δ*rpb5*). By applying a random mutagenesis approach we found that the amino acid exchange E197K in the C-terminal domain of the chimeric Rp5H, either alone or with additional exchanges in the N-terminal domain, leads to heterospecific complementation of the growth deficiency of Δ*rpb5*. Moreover, using a recently described genetic system for *Pyrococcus* we could demonstrate that the corresponding exchange E62K in the archaeal RpoH subunit alone without the eukaryotic N-terminal extension was stable, and growth experiments indicated no obvious impairment *in vivo*. *In vitro* transcription experiments with purified RNA polymerases showed an identical activity of the wild type and the mutant *Pyrococcus* RNA polymerase. A multiple alignment of RpoH sequences demonstrated that E62 is present in only a few archaeal species, whereas the great majority of sequences within archaea and eukarya contain a positively charged amino acid at this position. The crystal structures of the *Sulfolobus* and yeast RNA polymerases show that the positively charged arginine residues in subunits RpoH and Rpb5 most likely form salt bridges with negatively charged residues from subunit RpoK and Rpb1, respectively. A similar salt bridge might stabilize the interaction of Rp5H-E197K with a neighboring subunit of yeast RNA polymerase and thus lead to complementation of Δ*rpb5*.

## Introduction

Archaea contain only one multi-subunit RNA polymerase (RNAP), whereas eukarya contain at least three RNAPs with different functions. Nevertheless, the overall structure of the archaeal and the eukaryal RNAPs is very similar and consists of about 11 or 12 subunits [1]–[3]. Based on sequence and structural comparison, the subunits of the RNAPs of all domains of life can be divided into three classes [2], [4]. Class I subunits are conserved in all three domains whereas class III subunits are unique to each domain. Class II subunits are only present in eukaryal and archaeal RNAPs, but not in the bacterial domain. The following five eukaryal subunits with the corresponding archaeal subunits in brackets are the representatives of this class: Rpb5 (RpoH), Rpb6 (RpoK), Rpb8 (RpoG), Rpb10 (RpoN) and Rpb12 (RpoP) [2], [4]. It is interesting to note that subunit RpoG was identified only recently and seems to be present only in crenarchaeota but not in euryarchaeota [3], [5]–[7].

The individual structures of these class II subunits as well as their interactions with other subunits are highly conserved between archaea and eukarya [6], [8]. However, the eukaryal Rpb5 and the archaeal RpoH show a major difference in size [9], [10]: The N-terminal domain of Rpb5 is missing in RpoH whereas the C-terminal domain is conserved between both subunits [9]. The N-terminal domain marks the far end of the DNA channel of the eukaryal RNAP subunit and probably accounts for the Rpb5/DNA contacts found ahead of the transcription fork in RNAP II [1], [11], [12]. This exposed position enables interaction with different transcription factors, for instance Rpa30 of the TFIIF complex or protein X of the hepatitis B virus [13]–[15]. The C-terminal domain of Rpb5 is in close contact with Rpb1 as indicated by the structure of RNAP II [1]. Yeast two hybrid experiments also demonstrated an interaction with Rpa190 and Rpc160, the corresponding RNAP I and III subunits [16], [17]. Although the corresponding large RNAP subunit is split in archaea into two genes encoding subunits A′ and A″ [18], a strong interaction of A″ with RpoH has been demonstrated by Far-Western blot experiments with *Pyrococcus* (*P.*) *furiosus* components and with structural data from the *Sulfolobus* (*S.*) *solfataricus* RNAP [2], [8].

In archaea it is possible to reconstitute an active RNAP from individual subunits [19], [20], allowing for the functional analysis of individual subunits. Using an *in vitro* reconstituted archaeal RNAP lacking RpoP has revealed that this subunit is important for open complex formation and that the corresponding yeast Rpb12 subunit was able to rescue the *in vitro* activity of the ΔP enzyme [21]. Vice versa, yeast genetic experiments demonstrated that *Pyrococcus rpoP* under the control of a strong yeast promoter could complement the lethal phenotype of a *rpb12* deletion mutant [21]. Similar experiments using a reconstituted enzyme lacking subunit H showed that this subunit is also important for open complex formation as well as initial transcription, and yeast Rpb5 was able to rescue the *in vitro* activity of a reconstituted ΔH enzyme [22]. In contrast to the *in vitro* results with Rpb5 and the successful complementation experiments with *rpoP*, a chimeric *rp5H* construct encoding the N-terminal yeast domain and the C-terminal domain from *P. furiosus* is unable to complement the lethal phenotype of a yeast *rpb5* deletion strain [23 and unpublished data]. This negative complementation result in yeast with the archaeal RpoH domain is also in line with Rpb5 complementation experiments with the human counterpart. Rpb5 is the only common subunit in yeast which could not be substituted with the human homologue [24], [25]. Only the C-terminal part of human Rpb5 could complement the corresponding sequence in yeast. To identify those amino acids which seem to be responsible for the negative complementation result, a more detailed mutational analysis was performed. This experiment revealed that only a small central segment (positions 121–146 in *Saccharomyces cerevisiae*) within the N-terminal domain could not be substituted with the corresponding human sequence [26]. These results prompted us to take a similar approach with the chimeric Rp5H construct. Using a combination of random mutagenesis and library selection in the Δ*rpb5* strain we looked for mutants which activate the chimeric *rp5H* construct. We found that a single amino acid exchange within the archaeal domain of Rp5H is sufficient for complementation. Furthermore, *in vivo* and *in vitro* experiments with *P. furiosus* showed that this single exchange within RpoH does not influence the activity of the archaeal RNAP.

## Materials and Methods

### Strains, plasmids and general DNA manipulations

Strains and plasmids used in this study are listed in Table 1. Restriction enzymes and DNA polymerases for PCR reactions were purchased from NEB (Ipswich, USA). Genomic DNA was isolated using the ReliaPrep™ Kit from Promega (Mannheim, Germany).

**Table 1:** Strains and plasmids used in this study.

Name	Genotype or description	Source or reference
**Strains**		
*Saccharomyces cerevisiae* YFN2	*MATa ade2-1 lys2–801 ura3–52 trp1*-Δ*63 his3*- Δ*200 leu2*-Δ*1 rpb5-* Δ::*URA3*::*LEU2/pPL44-RPB5*	[31]
*Thermococcus kodakarensis* KU216	Δ*pyrF*	[45]
*Pyrococcus furiosus*	Wild type	DSMZ 3638
*Pyrococcus furiosus* MUR38Pf	RpoH with an N-terminal His_6_ tag	This work
*Pyrococcus furiosus* MUR39Pf	RpoH with an N-terminal His_6_ tag and the E62K exchange	This work
**Plasmids**		
pRS423_*rpb5*	Rpb5 with an N-terminal Flag tag and the strong constitutive *RPS28B* promoter [34].	This work
pRS423_*rp5H*	Substitution of the conserved C-terminal Rpb5 domain with the corresponding region of RpoH	This work
pMUR27	Enables introduction of the N-terminal His_6_ tag of RpoH	This work
pMUR28	Enables introduction of the N-terminal His_6_ tag and the E62K exchange of RpoH	This work
pMUR43	Similar to pMUR27 but with the N-terminal Rpb5 domain in addition	This work
pMUR54	Similar to pMUR28 but with the N-terminal Rpb5 domain in addition	This work

### Cloning of the chimeric construct *rp5H*

The chimeric construct *rp5H* was generated by overlap-extension PCR [27] linking a DNA fragment encoding the N-terminal domain of Rpb5 (GenBank accession number NM_001178502; bp 1 - 444) with *rpoH* of *P. furiosus* (PF1565). The oligonucleotides 5′rpb5_EcoRI, 3′rpb5_rpoH, 5′rpb5_rpoH and 3′rpoH_Not (sequences are shown as Table S1) were used for PCR amplification, digested with *EcoR*I and *Not*I, and ligated into the predigested *E. coli*-yeast-shuttle-vector pRS423 [28]. The resulting construct was termed pRS423_*rp5H*.

### Generation of *rp5H* gene library

The *rp5H* gene library was generated using error-prone PCR as described previously [29], [30], with the following modifications: The PCR cycling parameters were 95°C for 5 min; 35 cycles at 95°C for 45 s, 55°C for 55 s, 72°C for 40 s, followed by 72°C for 10 min. The PCR mixture contained 1.0 mM MgCl_2_, 1.0 mM MnCl_2_, 0.35 mM dATP, 1.35 mM dTTP, 0.4 mM dCTP, 0.2 mM dGTP, 1 µM of each primer (5′rpb5_EcoRI and 3′rpoH_NotI), 5 U DNA polymerase (GoTag, Promega, Mannheim, Germany), and 25 ng of plasmid pRS423_*rp5H* as template. The resulting gene repertoire was ligated into pRS423 and used to transform electrocompetent *E. coli* XL1 Blue MRF' cells (Life Technologies GmbH, former Stratagene, Darmstadt, Germany). Colonies grown on SOB rich medium agar containing 20 mM glucose and 150 µg/ml ampicillin were swept off the plates, followed by the isolation of the plasmid library containing the *rp5H* mutants. As estimated from the number of grown colonies and the ligation efficiency tested by colony PCR, the library contained approximately 4.5×10^5^ independent mutants. Sequencing of 10 clones revealed an average number of 11+/−7 nucleotide exchanges per gene, with a minimum number of two and a maximum number of 22 base substitutions. A bias for AT (71%) over GC (29%) exchanges and a ratio of transitions over transversion of 0.63 were observed.

### Transformation and *in vivo* complementation of *Saccharomyces cerevisiae* YFN2

For complementation assays the yeast strain YFN2 was used [31]. Plasmid DNA from the gene library was used to transform YFN2 cells carrying a chromosomal deletion of *rpb*5 rescued by an *rpb5/URA3* plasmid (pPL44-*rpb5*). The transformed Δ*rpb5* cells were streaked out on synthetic dropout agar plates (without histidine and uracil) and incubated for three days at 30°C. In order to identify *rp5H* variants that could complement for *rpb5 in vivo*, the 4.6×10^4^ grown cells were replica plated on synthetic dropout medium (without histidine) containing 5-fluoroorotic acid (FOA; 1 gL^−1^; Toronto Research Chemicals, North York, Canada) and incubated up to ten days at 30°C [32]. Plasmid DNA was isolated from 104 single colonies and used to retransform Δ*rpb5* cells, which were again plated on minimal media containing FOA. Out of the 53 positive colonies that grew after retransformation (true positive clones), the inserts of 12 individual clones were sequenced to identify amino acid exchanges allowing for complementation. All but one of the isolated pRS423_*rp5H* plasmids contained an *rp5H* insert coding for the E197K exchange, either alone or together with various combinations of other substitutions. The fastest growing colony contained an *rp5H* insert coding for the V23I+S68Y+M75K+E197K exchanges. The latter exchanges were cloned in different combinations in the background of the E197K exchange using megaprimer-PCR [33].

### Constructs for the *P. furiosus* mutants

To perform selection for the *Pyrococcus* transformants with the antibiotic simvastatin, a resistance cassette was assembled using overlap extension PCR reactions [27]. It consists of the promoter region (−250 to −1) of the *glutamate dehydrogenase* gene (*gdh*, TK1432; primers SimR-Prom1-F and TK0914TK1431Pr-R), the coding region of the *hydroxymethylglutaryl CoA* reductase (TK0914; primers: TK0914-F and TK0914-R), and a sequence for termination of transcription downstream of the *gdh* gene (TK1432; primers: TK0914TK1431T-F and SimR-Term2-R) from *Thermococcus kodakarensis*. The fused DNA fragments were inserted into a *Sma*I hydrolyzed pUC19 vector by blunt end ligation.

Plasmid pMUR27 was created to introduce a hexahistidine/streptavidin tag at the N-terminal region of the RNAP subunit RpoH for purification. For the construct, the following four fragments were combined: An upstream region of PF1565 (primers Hup-ERV-F and Fus-Hup-tkR-R), the *rpoH* gene and the downstream region of PF1565 (primers Fus-Strep-H-F and H-dwn-ERV-R), the resistance cassette mentioned above for the selection with simvastatin (primers SimR-Prom1-F and Fus-SimR-pr-R) and the transcriptional promoter of *rpoH* (primers H-Prom-F and Fus-His-HPr-R). The purification tags were inserted by additional amplification of the *rpoH* fragment with an expanded forward primer (Fus-His-Strep-F). The fusion product was hydrolyzed with *Eco*RV and ligated into the *Sma*I site of pUC19 to establish pMUR27. This plasmid was employed to construct pMUR28 by introducing the single exchange E62K using the phosphorylated primers pf1565-Mut-E62K-F and pf1565-Mut-E62K-R.

The plasmids pMUR54 and pMUR43 are similar to pMUR27 and pMUR28, but also contain the DNA fragment encoding the N-terminal Rpb5 domain from yeast. This DNA fragment was amplified using the primers Rpb5-N-Domäne F and Rpb5-N-Domäne R and plasmid pRS423_*rp5H*(E197K) as template DNA. All DNA constructs were verified by sequencing.

### Transformation of *P. furiosus*

Standard heat shock transformation of *P. furiosus* with linearized plasmid DNA was performed as described previously [34]. Transformants were enriched with 10 µM simvastatin in SME-starch liquid medium at 85°C for 48 h and pure cultures were isolated after plating the cells on solidified medium in the presence of 10 µM simvastatin. The integration of the DNA fragments into the genome by double cross-over was verified by analyzing corresponding PCR products.

### Growth conditions for *P. furiosus* strains

*P. furiosus* was cultivated under anaerobic conditions at 85°C–95°C in SME medium with 0.1% starch, yeast extract and peptone, as described previously [34], [35]. For solidification, gelrite was added to a final concentration of 1%. The antibiotic simvastatin (Toronto Research Inc., North York, Canada) was dissolved in ethanol and sterilized by filtration. For a more detailed analysis of the growth behaviour of *Pyrococcus* strains with modified *rpoH* genes, bottle flasks with 20 ml ½ SME-starch medium (supplemented with 10 µM simvastatin) were used and incubated at 95°C. Cell numbers were analyzed with a Thoma counting chamber (0.02-mm depth; Marienfeld, Lauda-Königshofen, Germany) using phase-contrast microscopy. All experiments were done in triplicate and the statistical mean value was used for plotting.

### Purification of RpoH-Strep/His_6_ tagged RNAP and *in vitro* transcription

Purification of RNAP containing a His_6_ tag at the N-terminus of subunit H was performed as described previously for RNAP tagged at subunit D [34]. Cell extracts containing 20 mM imidazole were applied to NiNTA chromatography (1 ml His-Trap HP, GE Healthcare Europe GmbH, Freiburg, Germany), and bound protein was eluted with a 300 mM imidazole elution buffer. Further purification and desalting was obtained by gel filtration chromatography using a Superdex 200 column, equilibrated with 40 mM HEPES, pH 7.3, 250 mM KCl, 2.5 mM MgCl_2_, 0.5 mM EDTA, and 20% glycerol. Fractions were analysed for RNAP activity using *in vitro* transcription and SDS-PAGE analysis. *In vitro* transcription reactions were performed as described previously [36].

## Results

### The chimeric construct Rp5H is unable to complement a Δ*rpb5* strain

**Figure 1:**
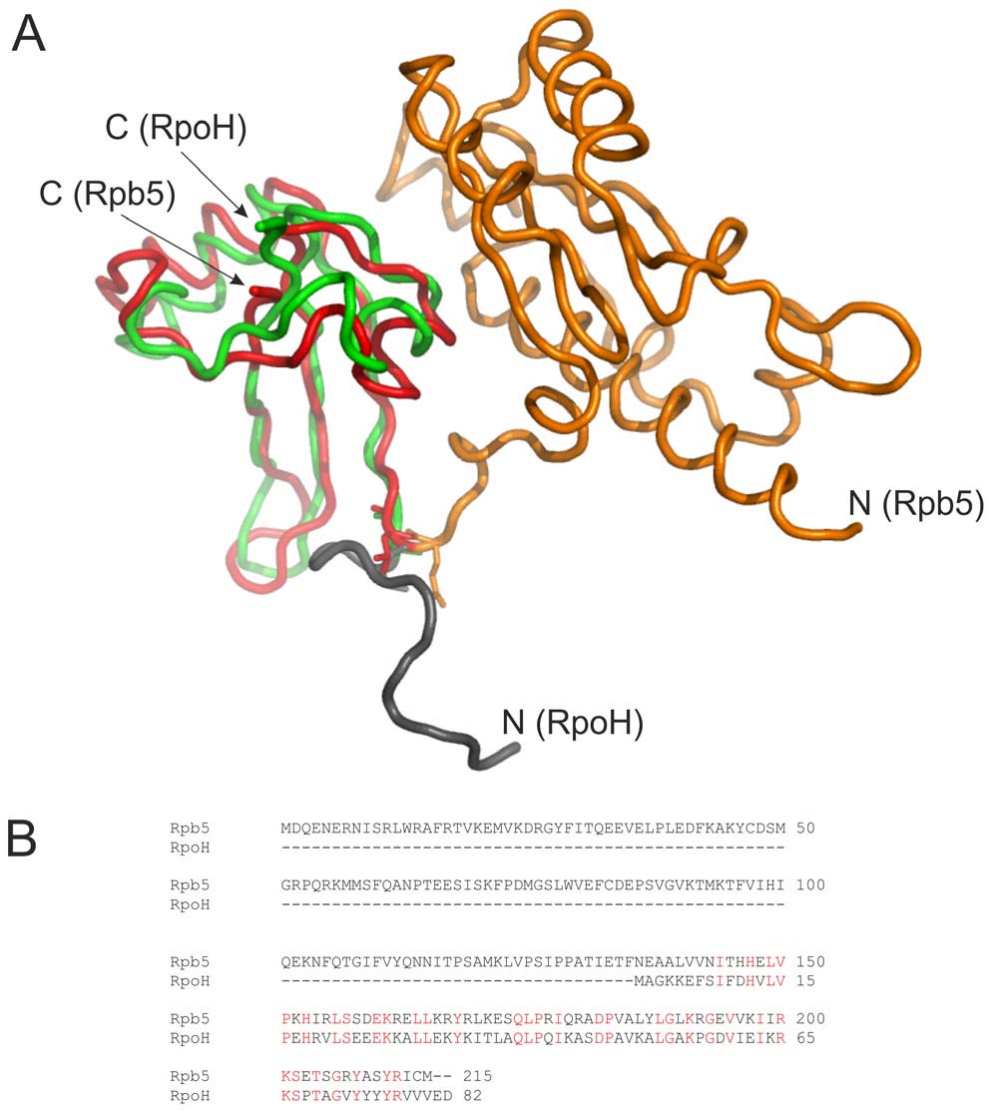
Superimposition of the structure and sequence alignment of Rpb5 from *S. cerevisiae* and RpoH from *P. furiosus*. A: The N-terminal part of Rpb5 is shown in orange and the C-terminal part in red [1]. The RpoH model in green was obtained using the program Modeller in combination with the solved RpoH structures of *Methanocaldococcus jannaschii*, *Methanobacterium thermoautotrophicus* and *S. solfataricus* [2], [9], [44]. For superpositioning the program DaliLite (www.ebi.ac.uk/Tools/structure/dalilite) was used. The amino acids at the N-terminus of RpoH which do not fit with the Rpb5 structure are shown in grey. The N-termini and the C-termini of Rpb5 and RpoH are labeled. B: The Rpb5 sequence of yeast and the RpoH sequence of *P. furiosus* were analyzed using a webserver of the program ClustalW2 (www.ebi.ac.uk/Tools/msa/clustalw2). Conserved amino acids are shown in red colour.

**Figure 2:**
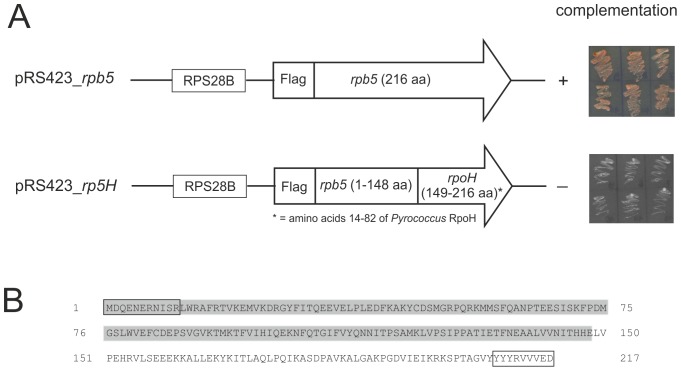
Complementation of the Δ*rpb5* strain. A: Schematic drawing of the constructs used for complementation. Plasmid pRS423_*rpb5* contains the wild type sequence as a positive control. The chimeric construct pRS423_*rp5H* codes for the N-terminal region of Rpb5 and the C-terminal region of RpoH from *P. furiosus*. Transformants were cultivated on synthetic dropout plates (without histidine) containing FOA and incubated at 30°C for 10 days. B: Amino acid sequence of the chimeric Rp5H construct without the Flag tag at the N-terminus. The N-terminal domain of Rpb5 is shadowed in grey followed by the C-terminal RpoH domain. The sequences excluded from random mutagenesis are boxed.

Fig. 1 illustrates the modular organization of Rpb5 consisting of a N- and a C-terminal domain. The N-terminal domain (orange part) is found only in eukarya, whereas the highly conserved C-terminal domain (red part) is also found in archaea (Fig. 1A). A superimposition using the structure of *Saccharomyces cerevisiae* and a model of *P. furiosus* (green part) shows a high similarity of the C-terminal domain between the yeast structure and the *Pyrococcus* model (Fig. 1A). Only the first few amino acids of the N-terminal region of RpoH do not fit into the superimposition (grey part). An alignment between both sequences confirmed the high degree of similarity of RpoH with the C-terminal domain of Rpb5 (Fig. 1B). These findings prompted us to reanalyze in more detail if this sequence and structural conservation enables a functional replacement of the C-terminal domain of Rpb5 with the corresponding archaeal domain. We used the yeast strain YFN2 with a chromosomal deletion of *rpb5* [31]. This strain carries the *rpb5* gene on plasmid pPL44-*RPB5* encoding the *URA3* marker. For complementation of the yeast strain in the presence of FOA we designed the shuttle vectors pRS423_*rpb5* and pRS423_*rp5H* (Fig. 2A). The positive control pRS423_*rpb5* contains Rpb5 with an N-terminal Flag tag and the strong constitutive *RPS28B* promoter [37]. In construct pRS423_*rp5H* the conserved C-terminal domain of Rpb5 was substituted with the corresponding region of RpoH without the first amino acids of the N-terminal region (Fig. 1A, grey part). The results of the complementation experiments indicated that the positive control could complement the Δ*rpb5* strain, but not the chimeric construct Rp5H with the archaeal RpoH domain replacing the yeast C-terminal domain (Fig. 2A). These results confirmed previous experiments [23 and unpublished data].

### A single amino acid exchange within RpoH of the chimeric construct Rp5H enables complementation

To identify amino acid exchanges in the chimeric Rp5H construct that lead to complementation, we undertook a random mutagenesis approach using error-prone PCR in the presence of MnCl_2_. As we used specific PCR primers corresponding to the first eleven and the last nine amino acids of Rp5H these regions were excluded from mutagenesis (Fig. 2B). Amplified gene products were ligated into the shuttle plasmid pRS423 and propagated in *E. coli*. The analysis of the gene library revealed about 4.5×10^5^ individual clones containing an average of 11 nucleotide exchanges.

**Figure 3:**
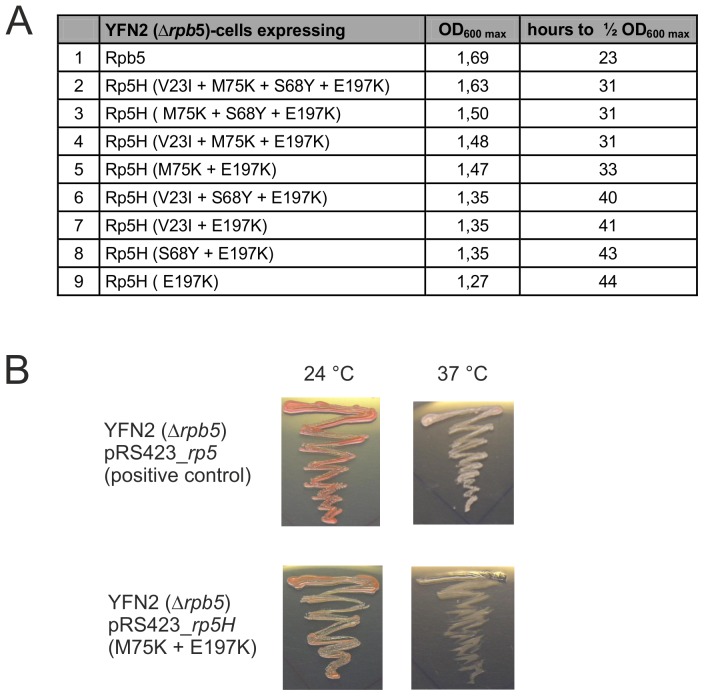
A single exchange within a chimeric Rp5H subunit can complement the loss of Rpb5 in yeast. A: Overview about the analyzed mutants and data on growth behavior. The mutants were grown in liquid culture in synthetic drop-out medium without histidine at 24°C. The maximum OD_600_ and the time needed to grow to half of the maximum OD_600_ are indicated in the table. B: Growth comparison of Δ*rpb5* transformed with the positive control pRS423_*rpb5* and the pRS423_*rp5H* (M75K + E197K) mutant on synthetic dropout plates (without histidine) containing FOA at 24°C and 37°C after five days of incubation.

The plasmid library was used to transform the yeast strain YFN2 with the *rpb5* deletion (Δ*rpb5*), and functional Rp5H variants were identified by the ability of their host cells to grow on synthetic dropout medium (without histidine) plates containing FOA. Positive clones were confirmed by retransformation and reassessed by *in vivo* complementation of Δ*rpb5*. Sequencing of 12 clones showed that all contained the exchange E197K in the C-terminal domain (stemming from RpoH) either alone or in combination with various exchanges in the N-terminal domain (stemming from Rpb5). The fastest growing colony contained an *rp5H* insert encoding the E197K exchange plus the V23I, S68Y, and M75K substitutions. In order to elucidate the effect of these N-terminal exchanges for complementation, they were cloned in isolation and in different combinations, always in the background of the E197K exchange (Fig. 3A). Following the transformation of Δ*rpb5* with the different pRS423_*rpb5* constructs, the growth of single colonies was compared in liquid minimal medium (synthetic dropout medium without histidine). Comparison of the growth behavior of the yeast strain with wild type Rpb5 and the strain with the single exchange E197K revealed that the maximum growth was reduced from OD_600_  = 1.67 for the wild type to OD_600_  = 1.27 for the mutant; the time needed to reach 50% of the maximum OD_600_ value was increased from 23 to 44 hours for the mutant (Fig. 3A, compare lanes 1 and 9). The obvious growth disadvantage of the mutant containing the E197K exchange in comparison to wild type was reduced in the presence of additional substitutions in the eukaryotic-specific N-terminal domain (Fig. 3A). These exchanges (V23I, S68Y, M75K) stimulated the growth of the mutant containing E197K in an additive manner with M75K having the strongest influence. Growth experiments on solid medium at 24°C and 37°C confirmed the reduced growth rate of the Rp5H mutants compared to wild-type Rpb5 especially at higher temperatures (Fig. 3B and data not shown). However, taken together, using a random mutagenesis approach it was possible to identify a single amino acid exchange within the archaeal domain of the chimeric construct Rp5H consisting of the N-terminal Rpb5 domain from yeast and the C-terminal archaeal RpoH domain which enables heterospecific complementation of the Δ*rpb5* yeast strain.

### The single E62K exchange within RpoH has no influence on the growth behavior of *P. furiosus*

**Figure 4:**
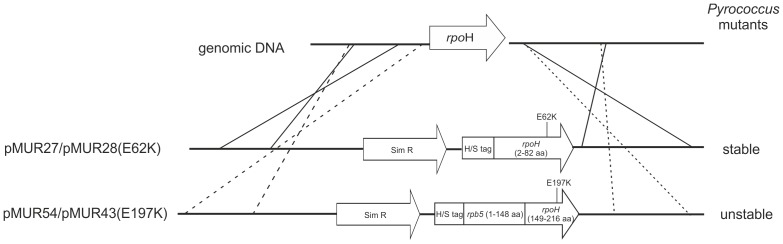
Schematic drawing of the plasmids used for the construction of the *Pyrococcus* mutants. The plasmids were assembled by overlapping PCR. Plasmid pMUR27 contains the wild type sequence of RpoH and plasmid pMUR28 comprises RpoH with the single exchange E62K. Plasmids pMUR54 and pMUR43 contain the chimeric constructs. SimR is an additional copy of the *hydroxymethylglutaryl CoA reductase* from *Thermococcus kodakarensis* (TK0914) and provides resistance against simvastatin. For purification, a His_6_ and a Strep tag is available at the N-terminal region of all constructs. At the right side of the figure the stability of the corresponding *Pyrococcus* mutants is indicated.

To investigate if the identified exchange of a negatively for a positively charged residue influences the growth behavior of *P. furiosus* in the background of Rp5H and RpoH we used a recently developed genetic system for *P. furiosus* [34], [35]. The plasmids pMUR27, pMUR28, pMUR43 and pMUR54 were used to establish the corresponding mutants within the *Pyrococcus* genome (Fig. 4). The plasmid pMUR28 is identical to pMUR27 except for the exchange E62K. This exchange is equivalent to the E197K exchange in the chimeric construct (Fig. 2). In the chimeric constructs pMUR54 and pMUR43 the eukaryotic N-terminal domain was fused with wild type RpoH or with RpoH-E62K (Fig. 4). For integration into the genome, the plasmids contain additionally 1000 bp upstream and downstream sequences of *rpoH* to enable double cross-over into homologous regions. For the selection of transformants a copy of the *hmg-CoA reductase* from *Thermococcus kodakarensis* (TK0914) was located upstream of the *rpoH* gene (Fig. 4). This gene provides resistance to the antibiotic simvastatin [34], [38]. For purification of the corresponding RNAP an additional His_6_/Strep tag was fused to the N-terminal region of RpoH.

After sequence verification the plasmids were linearized and used for transformation of *P. furiosus*. Characterization of the transformants using PCR demonstrated that the *Pyrococcus* mutants with the chimeric constructs were unstable. A more detailed analysis using different PCR amplicons revealed that these mutants rapidly lost the eukaryal N-terminal domain. After about 40 generations the N-terminal domain was no longer detectable whereas the selection marker was still present (data not shown). In contrast, the *Pyrococcus* mutant with the RpoH-E62K subunit was stable. Growth experiments at 95°C with the wild type construct of RpoH MUR38Pf and the mutant MUR39Pf with the exchange showed a similar growth behavior than the wild type (data not shown).

### *In vitro* transcription experiments indicate identical activities between wild type and mutated RNAP

**Figure 5:**
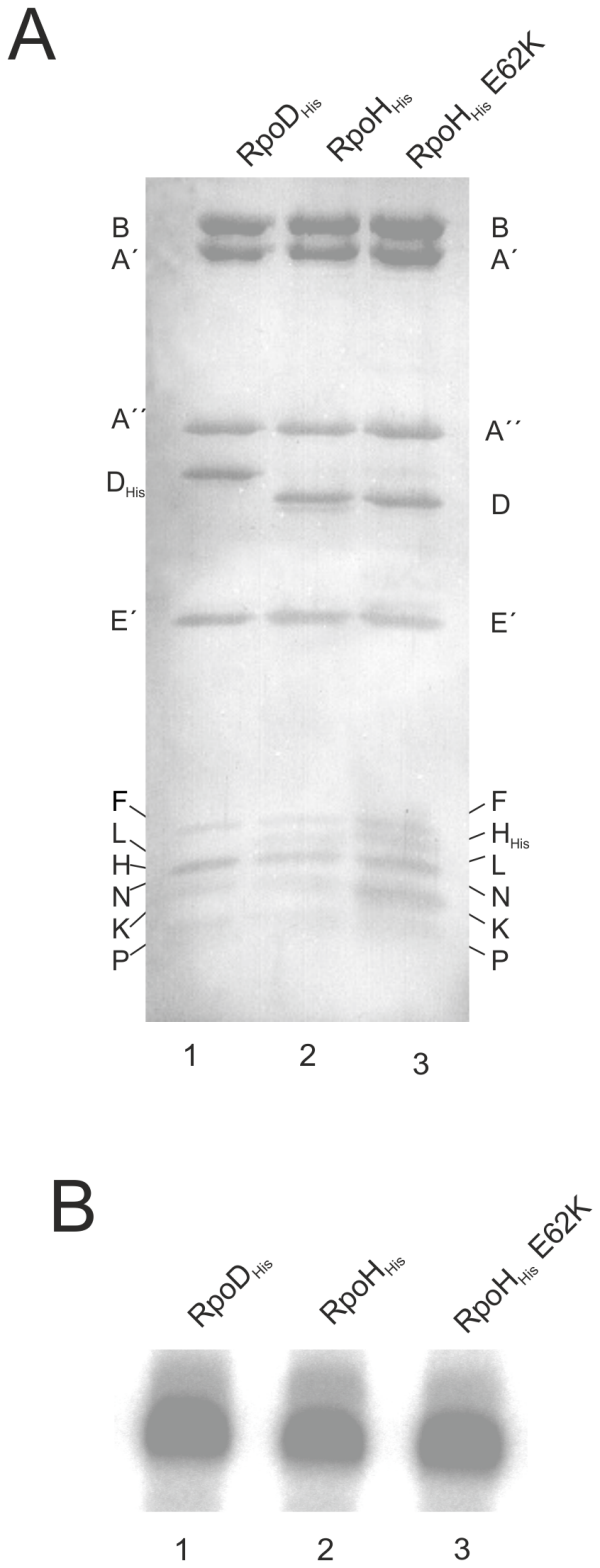
Purification and functional analysis of the RNAP. A: Silver-stained 10 to 20% gradient Tris Tricine SDS gel. Each lane contains 3 µg of purified RNAP. The analyzed RNAP fractions were purified using a His_6_ tag at subunit D (lane 1) or subunit H (lanes 2 and 3). B: *In vitro* transcription with the purified RNAP fractions. Identical amounts of RNAPs were used to transcribe the *gdh* template in the presence of the archaeal transcription factors TBP and TFB. The transcription assays were performed as described previously [36].

Although the growth analysis of RpoH wild type and RpoH-E62K indicated no phenotypical difference, we additionally performed an *in vitro* transcription analysis to compare the activities of the purified RNAPs. To produce cell mass for RNAP purification we cultivated the wild type and the mutant strain in large scale (100 liter fermenter) and purified the RNAP as previously described [34]. Using this two-step procedure consisting of a Ni-NTA and a gel filtration chromatography, highly purified RNAP could be obtained. The degree of purification was compared by gradient SDS-Page and silver staining with purified RNAP with a His_6_ tag located at subunit D (Fig. 5A, lanes 1 to 3). Both types of RNAP show for the most part a similar pattern of subunit stoichiometry, except that the electrophoretic mobilities of the modified subunits D (lane 1) and H (lanes 2 and 3) were increased due to the presence of the His_6_ tags. The composition of the smaller subunits seems to be slightly modified in the case of the RNAP with RpoH-E62K, but the exact stoichiometry of these subunits was not analyzed in detail. Using identical amounts of purified RNAP, an *in vitro* transcription experiment revealed that both, RNAP with RpoH wild type sequence and the RNAP with the E62K exchange, show the same transcriptional activity as the RNAP with the His_6_ tag at the C-terminus of subunit D (Fig. 5B, lanes 1 to 3). Taken together, the *in vivo* and *in vitro* experiments demonstrated that the single point mutation E62K of subunit H had no significant influence on the activity of the RNAP from *P. furiosus*.

## Discussion

In this paper we describe a random mutagenesis approach which converts the chimeric Rp5H construct consisting of the N-terminal Rpb5 domain from yeast and the C-terminal archaeal RpoH domain from *P. furiosus* from a yeast complementation inactive to a complementation active form. Specifically, we show that the single exchange E197K in the archaeal domain of the chimera is sufficient to functionally replace subunit Rpb5 of yeast RNAP.

Furthermore, using a genetic system for *P. furiosus* we could demonstrate that the corresponding substitution E62K in the RpoH subunit alone without the N-terminal extension seems to have almost no effect *in vivo* on the growth of *P. furiosus*. The establishment of the chimeric Rp5H construct in the *Pyrococcus* genome was not possible (Fig. 4). This finding is in contrast to *in vitro* data with a reconstituted ΔH RNAP enzyme of *P. furiosus* [22]. In this case yeast Rpb5 was able to rescue the activity of the reconstituted ΔH enzyme. This indicates that the additional presence of the N-terminal region in the chimeric Rp5H construct did not cause RNAP instability due to space problems. Along the same lines, a superimposition of the crystal structure of a crenarchaeal RNAP with the cryo-electron microscopy structure of the euryarchaeal RNAP from *P. furiosus* exhibited an easily accessible region in this part of the RNAP [39]–[41]. Furthermore, in some crenarchaeota an additional subunit of the RNAP, named Rpo13, is located at this position [7], [39]. The most likely explanation for the failure to establish the chimeric construct in the *Pyrococcus* genome is the thermolabile nature of the N-terminal yeast domain. We assume that this part of the construct is sensitive to denaturation at the high growth temperature of *P. furiosus*, which might cause precipitation of the entire subunit. The *in vitro* experiment mentioned above was done at 70°C for 30 min [22] whereas the *in vivo* approach required long-term stability of the chimeric construct at 95°C.

**Figure 6:**
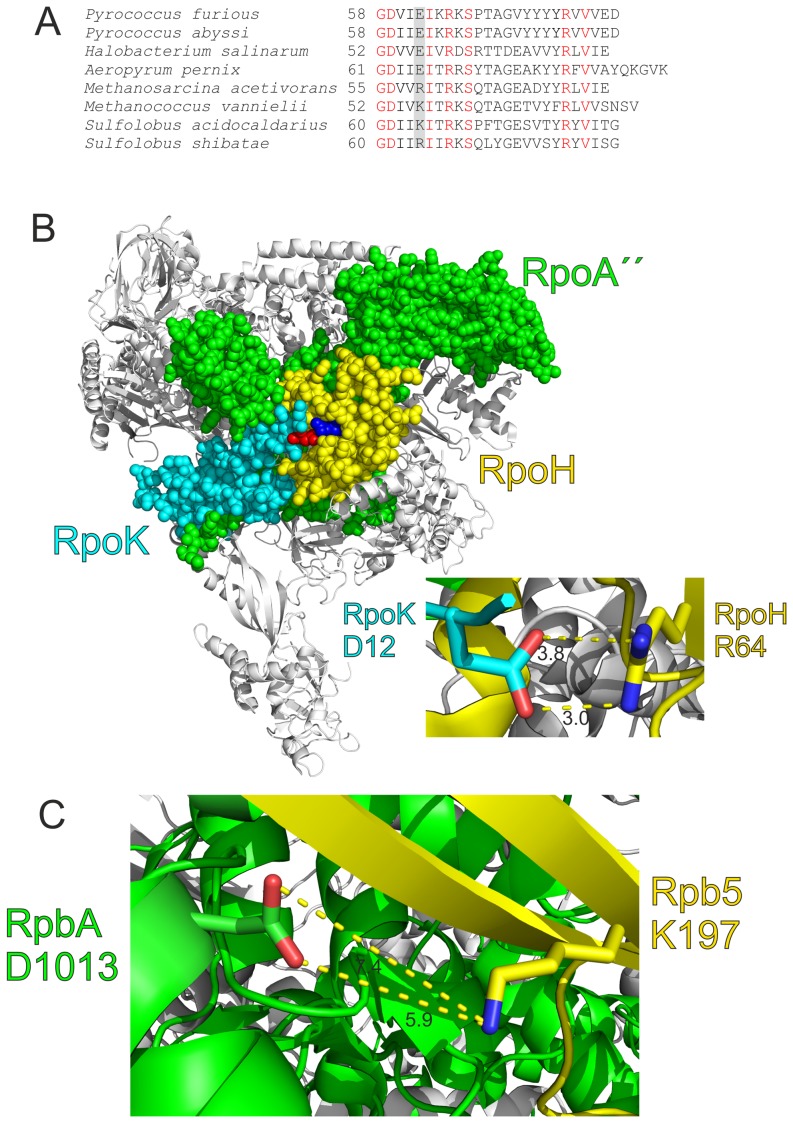
Sequence alignment and structural analysis of RpoH. A: Part of a multiple alignment of several RpoH sequences. For the alignment a webserver of the program ClustalW2 was used (www.ebi.ac.uk/Tools/msa/clustalw2). Conserved amino acids are shown in red and the positions corresponding to E62 of *P. furiosus* are shadowed in grey. B: Overall structure of the archaeal RNAP from *S. shibatae* (ID code in the Protein Data Bank: 2WAQ). Subunits A″, H and K are shown in space filling mode with different colors. Residue R64 (E62 in *P. furiosus*) of subunit H is shown in blue, and the putative salt bridge interaction partner D12 of subunit K is colored in red. In the close-up the distances between the nitrogen atoms of the arginine side chain and the oxygen atoms of the glutamate side chain are indicated in Å. The figures were drawn using the program PyMOL (www.pymol.org). C: Close-up of a putative salt bridge between K197 of Rpb5 and D1013 of RpbA from *S. cerevisiae* (ID code in the Protein Data Bank: 4BBR). The distances between the nitrogen atom of the lysine side chain and the oxygen atoms of the glutamate side chain are indicated in Å.

The most interesting result from our data is that the single amino acid exchange E197K in the archaeal RpoH domain is sufficient to activate the chimeric subunit for complementation in yeast. To put this finding into a broader context, we analyzed the corresponding regions of subunit RpoH in several archaeal species using a multiple sequence alignment (Fig. 6A). The results of the sequence comparison indicate that mainly three different amino acids are present at this position: The negatively charged amino acid E is only found in the order of *Thermococcales*, *Halobacterium* and *Aeropyrum pernix*, whereas the positively charged amino acids R and K are present in all other archaea, examples are shown from the *Sulfolobales* order and the methanogens (Fig. 6A). A detailed analysis of the corresponding position in the crystal structure of the *S. shibatae* RNAP revealed that R64 of subunit H most likely forms a salt bridge with D12 of subunit K (Fig. 6B; [3]). The distance between both amino acids is less than 4 Å, which is in perfect agreement with an ionic interaction (Fig. 6B, close-up; [41]). In this context it is interesting to note that the region containing D12 in subunit K of *S. shibatae* is missing in the K subunit of *Pyrococcus* (data not shown), which might allow for the negatively charged residue E62 in RpoH without generating electrostatic repulsion. As the crystal structure of the *Pyrococcus* RNAP is unavailable, a putative interaction partner of amino acid E62 is presently unknown.

At the position in subunit Rpb5 of Eukarya which corresponds to E62 in RpoH almost always a positively charged amino acid (R or K) is found (data not shown). The known structures of yeast RNAP contain only a part of Rpb6, which is the eukaryal subunit corresponding to RpoK [1], [43]. It is therefore not possible to decide whether the lysine residue in Rpb5 and, by inference the newly introduced K197 residue in Rp5H, is engaged in a salt bridge interaction with a negatively charged residue from Rpb6 as found for the RpoH-RpoK pair in *Sulfolobus* RNAP. A RNAP structure with the complete Rpb6 molecule would be required to answer this question. On the other side, it has been shown that the region around E197K of Rpb5 is in close contact with subunit Rpb1 [26]. A detailed analysis of the yeast RNAP structure identifies amino acid D1013 of Rpb1 as a putative interaction partner for amino acid K197 of Rpb5, although the distance between the nitrogen of the lysine and the oxygen of the aspartate with almost 6 Å is relatively large for an ionic interaction (Fig. 6C; [42], [43]). Nevertheless, the strong demand for a positive charge at this position of the Rp5H chimera for the formation of an active yeast RNAP indicates that this amino acid is most likely involved in the formation of a salt bridge either between D1013 of Rpb1 or with a negatively charged amino acid of subunit Rpb6 in a similar way found for the *Sulfolobus* RNAP. Therefore, the presented data clearly demonstrate that the approach of random mutagenesis and library selection is a suitable tool to identify important amino acids which play a role during the evolution of RNAPs from eukarya and archaea.

## Supporting Information

Table S1 Oligonucleotides used in this study.
(DOCX)
