# Supplementary material for: Activation of a Chimeric Rpb5/RpoH Subunit Using Library Selection
Source: PLoS One. 2014 Jan 29;9(1):e87485. doi: 10.1371/journal.pone.0087485 (PMC3906176; doi:10.1371/journal.pone.0087485)
Supplement: Table S1 — Oligonucleotides used in this study. (DOCX) [file pone.0087485.s001.docx]

**Supplementary Data**

**Table S1:** Oligonucleotides used for cloning, restriction sites are underlined.

| \| primer \| oligonucleotide Sequence (5’🡪3’) \| \| --- \| --- \| \| 5’rpb5_EcoRI \| GCTAAGACAGAATTCATGGACCAAGAAAATGAAAGAAACATCTCAAGA \| \| 3’rpb5_rpoH \| ATGTTCAGGTACTAATTCGTGGTGAGTAATA \| \| 5’rpoH_rpb5 \| TATTACTCACCACGAATTAGTACCTGAACAT \| \| 3’rpoH_NotI \| CCCCCGGCGGCCGCCTAGTCTTCAACAACAACCCTATAGTAG \| \| SimR-Prom1-F \| GGGCATTTCATCATTTTTATGAA \| \| TK0914TK1431Pr-R \| CTCAACAAGCTCTTCAAAGTTCATTCATACCACCTCATTTCGGTAAT \| \| TK0914-F \| ATGAACTTTGAAGAGCTTGTTGAGAAGGTAGC \| \| TK0914-R \| CTAACGTCCAAGCTCCTTATGGGC \| \| TK0914TK1431T-F \| CATAAGGAGCTTGGACGTTAGTTTCTTCTCCCTTTTCTCTTG \| \| SimR-Term2-R \| CGAAGGCTTAAACTTCTGTTTCG \| \| Hup-ERV-F \| GGACTTGATATCCCCAGGTCGC \| \| Fus-Hup-tkR-R 5´ \| AAATGATGAAATGCCCTTAGTCTCTAGTATATAAGCTTTACTCAACC \| \| Fus-Strep-H-F \| CACCCACAGTTCGAGAAGGCGGGGAAAAAGGAAT \| \| H-dwn-ERV-R \| CTCAACGATATCCTTATCTCTCTCC \| \| Fus-SimR-pr-R \| CTCAACCTTAATAAACATTATTAAAAACGAAGGCTTAAACTTC \| \| H-Prom-F \| TTTTTAATAATGTTTATTAAGGTTGAGTAAAGC \| \| Fus-His-HPr-R \| GTGATGGTGATGGTGATGCACGGAGAATTGCCCC \| \| Fus-His-Strep-F \| CATCACCATCACCATCACAGCGCATGGTCTCACC \| \| pf1565-Mut-E62K-F \| GAAATTAAAAGGAAAAGTCCAACT \| \| pf1565-Mut-E62K-R \| GATAACATCTCCCGGCTTAG \| \| Rpb5-N-Domäne F \| ATGGACCAAGAAAATGAAAGA \| \| Rpb5-N-Domäne R \| TTCGTGGTGAGTAATATTAACCA \| |
| --- | --- | --- | --- | --- | --- | --- | --- | --- | --- | --- | --- | --- | --- | --- | --- | --- | --- | --- | --- | --- | --- | --- | --- | --- | --- | --- | --- | --- | --- | --- | --- | --- | --- | --- | --- | --- | --- | --- | --- | --- | --- | --- | --- | --- | --- | --- |
